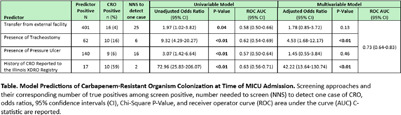# Comparison of Targeted Admission Screening Strategies for Carbapenem-Resistant Organisms (CROs)

**DOI:** 10.1017/ash.2025.422

**Published:** 2025-09-24

**Authors:** Tanner Shull, Michael Schoeny, Mary Hayden, William Trick, Michael Lin, Sarah Sansom

**Affiliations:** 1 Rush University Medical Center; 2 Rush University Medical Center; 3 Rush University Medical Center; 4 Cook County Health; 5 Rush University Medical Center; 6 Rush University Medical Center

## Abstract

**Background:** Admission screening for CRO carriage may prevent transmission, but there is a lack of consensus on the best targeted approach. Using a well-characterized cohort of medical intensive care unit (MICU) patients prospectively screened for CRO carriage at time of admission (MAriMbA cohort), we compared the effectiveness of common targeted strategies (singly and in combination) available to hospitals in Illinois to identify MICU patients at risk for CRO carriage, including: (a) screening patients transferred from external facilities (e.g., short- and long-term acute care hospitals); (b) screening patients with a tracheostomy or pressure ulcer; or (c) querying the Illinois XDRO registry for prior CRO history. **Methods:** Results of rectal swab samples collected within 48 hours of MICU admission during 1/2017-1/2018 and cultured for CROs (carbapenem-resistant Enterobacterales [CRE], CR Pseudomonas aeruginosa [CRPA], and CR Acinetobacter baumannii [CRAB]) were used as the reference standard. Patients’ status as direct transfer from an external healthcare facility and presence of tracheostomy or pressure ulcer were collected prospectively during the MAriMbA study. History of CRO colonization before MICU admission was queried retrospectively from the Illinois XDRO Registry (xdro.org), with the limitation that most reports available during the study period were restricted to CRE. We evaluated each predictors’ independent association with admission CRO status and combined variables in a planned logistic regression modeling approach. **Results:** CRO colonization was detected in 37 (2.6%; including 26 CRE, 10 CRPA, and 1 patient co-colonized with CRE and CRAB) of 1,423 unique MICU admissions. For univariate analyses, presence of a tracheostomy (OR 9.32, 95% CI 4.29-20.27), presence of pressure ulcer (OR 3.07, 95% CI 1.42-6.64), transfer from an external healthcare facility (OR 1.97, 95% CI 1.02-3.82), and prior CRO history reported to the Illinois XDRO Registry (OR 72.96, 95% CI 25.83-206.07) were associated with higher odds of CRO colonization. A model combining these variables improved the predictive capability (AUC 0.73) (Table). Prior CRO history reported to the Illinois XDRO Registry identified 27% of CRO cases, with number needed to screen (NNS) of only 2 patients. Adding tracheostomy, pressure ulcer, and external facility transfer together improved detection of admission CRO cases to 68%, with NNS of 20 patients (Figure). **Conclusion:** In a region with well-established inter-facility communication of CRO history via the Illinois XDRO Registry, the addition of screening patients with a tracheostomy, transfer from an external facility, or pressure ulcer may improve early identification of CRO carriage at time of MICU admission.

**Figure:**
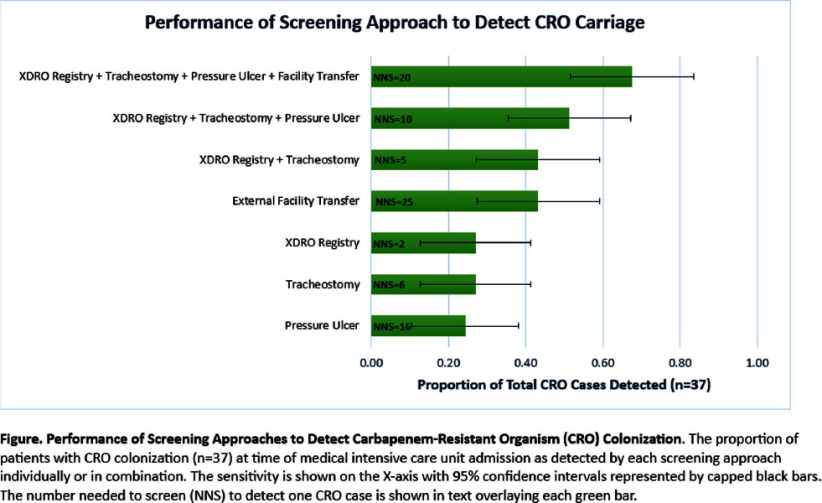

**Figure:**